# Evidence for Cross-Cultural Support for the Underdog: Is the Affiliation Driven by Fairness and Competence Assessments?

**DOI:** 10.3389/fpsyg.2018.02246

**Published:** 2018-11-19

**Authors:** Nadav Goldschmied, Yair Galily, Kenneth Keith

**Affiliations:** ^1^Department of Psychological Sciences, College of Arts and Sciences, University of San Diego, San Diego, CA, United States; ^2^Interdisciplinary Center Herzliya, Herzliya, Israel

**Keywords:** underdog support, fairness, competence, attributions, sport

## Abstract

Jesus told his disciples, “Truly I tell you, it is hard for someone who is rich to enter the kingdom of heaven. Again I tell you, it is easier for a camel to go through the eye of a needle than for someone who is rich to enter the kingdom of God.” (Matthew 19:23–24). Ditto for heroes. The current study suggests that “humble beginnings” is also a prerequisite for one to become an adulated entity. Participants from China, Israel, and Japan read of two sports teams with disparate expectations and/or financial resources about to face each other. Support was extended to the lesser one. When the two domains of comparison were contrasted, participants wished the lower resources/high expectations team to win the game. This finding was interpreted as an impetus to maintain basic fairness based on competency assessments, both fundamental and universal psychological needs, at the root of the choice to support underdogs. In conclusion, we explore how support underdog relates generally to the concept of heroism.

## Introduction

Edward Sagarin, the famed (and controversial) American sociologist opens his 1970 treatise “Who roots for the underdog?” with this sweeping statement “Everyone in America is for the underdog. How do we know? It is simple enough: Americans tell us that they root for the underdog.” (p. 425). In analyzing the writing of others, Sagarin determines “It is not a human trait but a specifically American trait, this sympathy for the underdog. In fact, sympathy goes with underdog like ham goes with eggs….” (p. 428). He associates underdog support with fighting the oppressor and arrives at the conclusion that underdog support is a uniquely American trait because of its democratic governing system. If this is indeed the case, one wonders if Sagarin is being somewhat US-centric by overlooking the fact that this same governing system is practiced in many other countries.

But even more fundamental questions arise from Sagarin’s writings. Are underdogs indeed supported by all, or at least the majority, of Americans? Sagarin only reviews the writings of other thinkers and does not trouble himself to test empirically the opinions of other Americans who are not members of the intellectual elite. If such an underdog effect does exist in the United States, is it exclusive to this country, as he proclaimed? Furthermore, is it based on the political system practiced in each country as he inferred, or is it actually a universal idea (ideal?) – a notion he vehemently rejects.

### Current Research Into Underdog Support

The defining feature of underdogs is the opposition they are facing. They struggle against entities, which are similar to themselves but significantly mightier. They often face another sport team or athlete (Vandello et al., 2007), a rival politician or business company (Goldschmied and Vandello, 2009; Paharia et al., 2010; Goldschmied et al., 2017), a neighboring country in a geopolitical struggle (Vandello et al., 2007), an opposing litigant (Songer and Sheehan, 1992) or another in their profession (Kim et al., 2008). As such, a numerical quantification of the disadvantage can be assessed, for example the likelihood to prevail in an upcoming competition (e.g., batting odds, expert predictions), resource availability [e.g., salaries, market share, donations to the cause (Bradley et al., 2018)], or past success (e.g., how did they fare against one another, how did they both fare against similar others?). We also contend that there are “code” terms or heuristics, as “mom and pop” operation, “humble beginnings” (Paharia et al., 2010) or “the little guy” (McGinnis and Gentry, 2009) which convey succinctly this significant disadvantage relative to the competition without requiring additional elaboration.

During the last decade or so empirical research has sustained Sagarin’s initial assertion that support and sympathy for underdogs is indeed a well-documented phenomenon in American culture (Vandello et al., 2007; Kim et al., 2008; Goldschmied and Vandello, 2009; Paharia et al., 2010; Goldschmied and Vandello, 2012). For example, Vandello et al. (2007) showed that when asked to make hypothetical predictions about a future Olympic competition, participants in the United States preferred countries with a less stellar past record of winning medals in the games over favorites with a superior record. Likewise, when United States participants read the same essay about the Israeli-Palestinian conflict, accompanied by a map that manipulated the relative size of the two sides (i.e., Palestinian territories framed as small compared to Israel vs. Israel being framed as small compared to its Arab neighbors in the Middle East), the responders defined the smaller entity on the map as the underdog, shifted support to it, and saw its position as more justified. Kim et al. used a more abstract manipulation, showing participants animated clips of round shapes rolling on either a flat surface or climbing uphill. In this study, participants liked the most a struggling ball (i.e., slowing down as if facing much adversity) when it was bumped “intentionally” by a mighty ball (which continued to roll at the same speed as if experiencing no difficulty) in comparison to a non-struggling ball or a struggling ball without a mighty adversary.

Once underdog support in the United States was demonstrated consistently, further research attempted to delineate the motivations behind the effect. Ideas such as the upward mobility bias (Davidai and Gilovich, 2015) demonstrate the considerable focus placed on the underdog’s motivation to rise up while the intentions of the favorite to suppress this effort are often overlooked. Alternatively, Goldschmied and Vandello (2012) argued for a memory bias rooted in the availability heuristic, such that when having to make a decision whether to support an underdog entity or not, past instances of underdog struggles are recalled. As underdog stories are prominently featured in those rare occasions when they overcome, people tend to overestimate the likelihood of future underdogs to do the same and this bias may contribute to the observed underdog support as winning is much cherished.

Another prominent explanation for underdog support revolves around fairness considerations. The rationale is that when significant differences in strength between direct competitors are highlighted, observers intuitively assume that the disparities are predicated on an unfair distribution of resources and that the underdog’s lesser endowment is not because of its own doing and shortcomings but due to the world being an unjust place. This attributional process moves them to extend their support to the “little guy” in the attempt to morally and psychologically rectify an unjust world. In support of this idea, Vandello et al. (2007) used a sport scenario in which the expectations of two unknown teams to win a future competition were manipulated independently from the amount of money the teams had at their disposal to spend on quality players (i.e., financial resources). As hypothesized, whenever a clear disadvantage was present (based on expectations, financial resources, or both), the lesser team was recognized as the underdog and was supported. However, support for the underdog was driven more by considerations of relative financial resources than by expectations of winning. Participants were less likely to see a team with relatively ample resources as an underdog even if the team had low expectations for success, compared to a team with lesser financial resources. With the loss of the underdog label, support was removed and extended to the stronger team. This shift in support tendencies seems due to a perceived lack of competence (i.e., despite an abundance of resources the team is not projected to do well). Stated differently, relative resources moderated the effect of expectations on liking and support, which was interpreted as evidence for fairness concerns based on attributional analysis at the root of underdog support tendencies.

### Underdog Support in the International Arena

The focus of the current investigation was an attempt to replicate Vandello et al. (2007) in exploring whether sympathy for lesser/underdog entities extends also to cultures outside the United States and whether this support is predicated similarly on fairness and competence assessments. We selected samples from three culturally diverse countries (Israel, Japan, and China), with the intention to serve three main goals. First, none of these countries has Anglo-Saxon origins, and thus may not share the same underlying values held by people in the United States who participated in the original study. Research studying political opinion polls shows underdog support in English-speaking countries such as the United Kingdom (Teer and Spence, 1974; Marsh, 1984), the United States (Fleitas, 1971; Ceci and Kain, 1982), and Australia (Goot, 2010), but data are not available beyond. Paharia et al. (2010), in the only past cross-cultural underdog support study, argued that cultural identity was indeed a moderator, as American college students preferred an underdog chocolate brand over a well-established competitor brand more so than their peers in Singapore. The researchers grounded their findings in the higher levels of individualism in the United States (Triandis, 1989; Markus and Kitayama, 1991), supposedly leading to higher appreciation for those who try hard and attempt to disrupt the regular order of business. They also contended the United States had a generally high regard for cultural idols who triumph over hardship or poverty as symbolized in the “American dream” narrative.

Second, past writers (Klapp and Heroes, 1962; Sagarin, 1970) were convinced that underdog support was uniquely American based on democratic values and education. Although this idea is somewhat anachronistic and contradictory in nature (as other countries also practice this governing system), we attempted to put this hypothesis to the test. In the current study two countries, Israel and Japan, share similar vibrant democratic political systems as the United States, while China is authoritarian, with single-party rule.

Third, all three sampled countries demonstrate substantial differences along the masculinity-femininity continuum based on Hofstede’s Cultural Dimensions taxonomy (Hofstede, 1980, 1991, 2001). Hofstede argued that when conducting cross-cultural comparison studies, researchers should consider five central dimensions: power distance, individualism vs. collectivism, short vs. long-term orientation, uncertainty avoidance, and masculinity vs. femininity. The last factor comprises the extent to which open conflict is tolerated and even encouraged in society. On the feminine side of the continuum, compromise and accommodation are valued. On the masculine end, a premium is placed on a show of force and assertiveness. Hofstede linked masculine orientation with support for the winner, while feminine orientation was associated with support for the underdog. Each country in the current investigation represents a unique standing along this continuum: Japan represents the most masculine oriented culture (in the 90 range based on the 100-point Hofstede scale), China scores similar to the world average (in the 50 range), while Israel tilts toward the feminine end (in the 40 range).

In the current investigation we then set out to determine how distinct cultures address how the “underdog” construct pertains to “resources” and “expectations” for “victory” and ascertain if there are cross-cultural differences in relating to the “underdog” construct accordingly. More specifically, we attempted to determine if there are cross-cultural differences in support for the lesser.

## Materials and Methods

### Participants

One hundred ten students from the Open University of Tel-Aviv in Israel (93 females, 17 males; mean age = 26.99, *SD* = 5.7), 107 students in China from Tianjin University of Sport (55 females, 52 males; mean age = 19.17, *SD* = 1.15) and 118 students from the Seirei Christopher University in Japan (82 women, 35 men, 1 unidentified; mean age = 19.73, *SD* = 1.93) completed the questionnaire.

### Procedure

Participants completed a one-page questionnaire in their classrooms. The first section presented participants with a brief sports scenario, including a request to imagine two teams about to play an important match (in an unspecified sport). We chose the context of sport because previous research has shown that the underdog label was strongly associated with this domain (Goldschmied, 2007; McGinnis and Gentry, 2009). We manipulated each team’s expectations and resources to produce four versions of the scenario. In the first version (expectations only), Team A was described by an unspecified source as having a 70% chance of victory versus 30% for Team B; in the second version (resources only), Team A was described as having a payroll of $100 million versus $35 million for Team B; in the third version (congruent expectations and resources), Team A was described as having both a 70% chance of victory and a $100 million payroll versus a 30% chance of victory and a $35 million payroll for Team B; and in the fourth version (incongruent expectations and resources) Team A had the greater chance of victory (70%) but a lower payroll ($35 million), compared to Team B (30% chance, $100 million payroll). Thus, the design was a 3 (country) × 4 (scenario) × 2 (team), with country and scenario being between-subjects factors and team a within-subjects factor.

As a manipulation check, participants were first asked to estimate which team was going to win the match (forced-choice). Next, they were asked how much they would like each team to win the game, on a 9-point scale ranging from 0 = “not at all” to 9 = “very much.” Finally, the participants were asked which of the two teams they would root for (forced-choice).

The research was approved by the internal review board of the University of South Florida.

## Results

### Who Will Win (Manipulation Check)

In the incongruent scenario, the less resources/high expectations team was defined as the favorite. Across all samples, about 85% believed that the stronger team would win the game. A chi-square test to assess the relation between scenario and prediction made was not statistically significant, χ^2^ (3, 332) = 4.43, *p* = 0.219. A chi-square test of the relation between country of origin and prediction made was significant, χ^2^ (2, 332) = 8.92, *p* = 0.012. Specifically, 76% of participants from China predicted that the top dog would prevail, as did 86% of Israelis and 91% of Japanese (all predictions at or above the baseline of 70% prediction used in the scenarios).

### Underdog Support

A three-way mixed model analysis of variance was conducted first to explore the effects of status (underdog vs. favorite) as a repeated-measure variable and the type of scenario and the country of the participants (between-subjects factors) on the support extended to the teams at play. In the incongruent scenario, the less resources/high expectations team was defined as the underdog entity based on the results of Vandello et al. (2007). Table 1 presents the mean ratings of desire for each team to win the game across the four conditions for each country. There was a main effect of status such that underdog entities were supported significantly more (*M* = 6.12) than favorites (*M* = 4.96), *F*(1,323) = 43.11, *p* < 0.000, partial eta squared = 0.118. In addition, there was a main effect of country, *F*(2,323) = 5.61, *p* = 0.004; partial eta squared = 0.034. *Post hoc* comparisons using Tukey HSD test indicated that participants from China (*M* = 5.75) and from Japan (*M* = 5.59) extended overall more support (to both sides) than participants from Israel (*M* = 5.28). There was also a main effect of scenario, *F*(3,323) = 2.99, *p* = 0.031; partial eta squared = 0.027. The resources-only condition generated more support (*M* = 5.81) than the expectations-only condition (*M* = 5.36), while the matching (*M* = 5.53) and non-matching (*M* = 5.46) scenarios were no different from all other conditions.

**Table 1:** Desire for each team to win the game as a function of expectations for success and resources, by country.

	Degree of liking to win (1–9)					

	Israel		China		Japan	
	Team A	Team B	Team A	Team B	Team A	Team B
Expectations only:						
Team A: 70% chance of victory, Team B: 30% chance of victory	5.06 (2.01)	5.77 (2.13)	4.34 (2.38)	6.31 (1.97)	4.65 (1.66)	5.90 (2.17)
Resources only:						
Team A: high payroll, Team B: low payroll	4.32 (1.4)	6.76 (1.92)	5.96 (2.01)	6.00 (2.08)	5.13 (1.11)	6.70 (1.66)
Expectations and resources congruent:						
Team A: 70% chance of victory + high payroll, Team B: 30% chance of victory + low payroll	4.81 (2.06)	5.46 (2.06)	5.15 (2.26)	6.81 (2.15)	5.11 (1.55)	5.86 (1.84)
Expectations and resources incongruent:						
Team A: 70% chance of victory + low payroll, Team B: 30% chance of victory + high payroll	5.46 (2.4)	4.63 (1.95)	6.35 (2.1)	4.96 (1.78)	6.10 (1.29)	5.28 (0.99)


In order to test the main hypothesis regarding whether underdog support is a cross-cultural phenomenon, a difference score was computed based on the support extended to the entity at a disadvantage minus the support for the team with an advantage across the four conditions (again, in the incongruent scenario the financial disadvantage was used as the anchor to determine which team was considered the underdog). This difference score was considered a reliable measure, as participants tended to extend support to one or the other of the teams but not to both at the same time, as evident by a negative correlation between supporting the underdog and supporting the favorite (*r* = –0.43, *p* < 0.01) [the interaction terms could not have been tested without computing a difference score, as averaging support would have washed out the differences extended to underdogs vs. top-dogs].

A two-way between-groups analysis of variance was conducted to explore the effect of country and type of scenario on the support extended to underdogs (a difference score). There was no effect of country on underdog support, *F*(2,323) = 0.036, *p* = 0.96. There was also no effect of scenario on underdog support, *F*(3,323) = 0.215, *p* = 0.87. Also, the interaction effect did not reach statistical significance [*F*(6,323) = 1.974, *p* = 0.07].

We also explored underdog support using a forced-choice format in which participants were asked to designate which team they would root for if they were present at the actual game. A chi-square test of the relation between scenario and team rooted for was not significant, χ^2^ (3,332) = 6.567, p = 0.09. Finally, underdog support was robust, as 52% of participants from Israel reported that they would root for the underdog, as well as 57% of Chinese and 72% of Japanese participants [vs. 68% in the American sample, Vandello et al. (2007)].

## Discussion

The current undertaking was a cross-cultural exploration in which Chinese, Japanese, and Israeli students were asked about their support for two competing sport teams with varying chances of success, financial resources, or both (with congruent and incongruent scenarios). Participants consistently extended their support to the lesser competitor, as did their American counterparts in the original study (Vandello et al., 2007). The results are telling in showing that fairness seems to drive support tendencies in a across-cultural manner (in 8 out of the 9 comparisons embodying a clear disadvantage, the lesser side was significantly supported and in none was the top-dog supported). Support was not conditioned upon benevolence or morality, as none of these elevated ethical qualities was stated or could be inferred from the minimal descriptions of the teams.

When the characteristics of the underdog team did not match (i.e., incongruent expectations and resources condition) participants in all three countries supported the entity with less financial resources (and higher expectations to win), again, similar to American participants (Vandello et al., 2007). The results are most informative in the incongruent condition as aptitude can be deduced. Competence thus seems to be a vital reason behind cross-cultural underdog support. Upon realizing that the team’s lower chances to prevail exist despite abundance of resources, loyalties shift and the low-expectations team is no longer supported. This finding based on the incongruent condition also sheds light on the expectations-only condition when underdog support was extended to the lesser team. It seems that without being fully informed, participants assume that the team’s diminished chances are based not on its own making but due to the “harsh” hand the team was dealt. Similarly, Paharia et al. (2010) reported that one essential dimension of the underdog label was “external disadvantage” rather than disadvantage altogether.

While we propose here a fairly systematic attributional process to determine competence at the root of the fairness assessment, caution must be practiced as our participants were not inquired directly about their thought process in making the decision whom to support. Past research (Dijksterhuis and Nordgren, 2006; Dijksterhuis et al., 2006) has found that not always do people engage in a deliberate-conscious decision-making process when deciding between options.

Indeed no cultural influences were detected in the three countries sampled at the macro level of analysis utilizing a difference score between the levels of support extended to each team. However, difference scores are often criticized as statistically conservative (Edwards, 1995; Jehn and Chatman, 2000) and thus it is worthwhile to study the data at a more granular level. For example, in the resources only manipulation, there seems to be no difference in the China data in rooting for either team. This country has gone through an unprecedented financial transformation in the recent times from a Communist society with government planning to one that is more market and consumer oriented and thus the psychological meaning of money in relating to others (Vohs et al., 2006) may still be in flux though this possible explanation is admittedly a *post hoc* one. Also, the effect of the expectations only manipulations seems less strong for Israel than in the other two countries. These manipulations and others should be considered in future cross-cultural underdog research as we operationalized “significant disadvantage” in merely two ways in the present investigation. Other untested variables denoting numerical disadvantage may, in turn, interact with culture to affect support for the underdog.

One question which emerges in light of this strong tendency to support the lesser is at what level of disparity this underdog support tendency emerges. As we did not manipulate the level of discrepancy in the current study we cannot determine when does the contrast between the competitors become perceptible enough to elicit underdog support (i.e., would a team with 45% chance to win as opposed to 30% in the present investigation engender the same support level?). Also of importance is whether the underdog support threshold is culturally dependent and whether once established it follows a linear trend or the disparity may become so great to bare that observers abandon the lesser eventually. Lastly, the methodology we utilized prevented us from teasing apart underdog support tendencies from schadenfreude or the desire to see the mighty fall (Feather, 1998; Feather and Sherman, 2002).

Because we find that support for the underdog is a cross-cultural phenomenon, we need to re-evaluate past research claims to the contrary. First, while Hofstede (1980, 1991, 2001) drew a clear association between cultures high on the femininity domain and underdog support, many questions arise with regard to both the reliability of his findings and the validity of his conclusions (McSweeney, 2002). Moreover, in achievement-related events, perceptions of fairness and attributions demonstrate somewhat similar patterns across cultures when the self is not implicated (Betancourt and Weiner, 1982; Berman et al., 1985). In addition, Hofstede’s definition of the underdog construct tends to be incomplete with regard to the hero worship he associates with cultures. For example, he contends that “In masculine cultures children learn to admire the strong: popular fiction heroes made in the United States include Batman and Rambo.” (Hofstede, 2001; p. 300). He neglects to mention that while Rambo is certainly a masculine figure, he is also an underdog, a lone wolf, fighting not only a mighty enemy but also his superiors. On the flip side, Hofstede argues that “In feminine cultures children learn sympathy for the underdog and the antihero. “Rasmus Klump” (called Petzi in translation), small and friendly, is a Danish comic hero…”. He neglects to mention that antiheros pervade American culture as well–such as Charlie Brown, Shrek and Garfield–and anecdotal evidence for underdog support in folklore, anime, horse racing, and film is apparent in all the cultures studied (Tsabari and Tzor, 1996; Associated Press [AP], 2004; Yu-Gi-Oh! Heart of the Underdog [Anime], 2004; Wong and Ding, 2008; Caplan, 2017).

### The Underdog as a Hero

Heroes, we know, should have some basic qualities to earn their coveted label. First and foremost, they need to be benevolent and engage in moral deeds (or at least engage in the call for others to do so) as well as be caring and compassionate (Eden et al., 2015).

Another important hero characteristic is the adversity they face. Heroes must be opposed by forces which are significantly mightier than themselves and are likely to retaliate against them when they pursue their noble causes (otherwise, they are more befitting the label of “do-gooders” rather than heroes). So paradoxically, heroes are defined by their opposition and the antagonism they encounter (Allison, 2017). The hostility or hardship they face, in turn, requires them to be competent in their pursuit. In the absence of competence, they are destined to fail as they face an uphill battle. The combination of all these dimensions brings about another important facet of heros: the inspiration and adulation they arise in us (Allison and Goethals, 2016).

Allison and Goethals (2011) asked participants to generate traits describing heroes. Based on factor and cluster-analytic statistical procedures, they identified eight general categories of hero traits. These traits are in-line with the heroes’ themes described above and consist of “selfless” and “caring” (i.e., benevolent), “smart,” “strong,” “charismatic,” “reliable” (i.e., competent), “resilient” (i.e., facing strong opposition), and “inspiring,” which was identified by a different group of participants as the most important dimension of being a hero (Allison and Goethals, 2011).

Underdogs are a subset class of heroes. They possess most of the positive characteristics associated with being a hero, but not necessarily all. They are certainly inspiring as we strongly root for them (Allison and Goethals, 2008; Kim et al., 2008). They are also perceived as competent based on the findings of the current study as well as Vandello et al. (2007). However, they are not necessarily more virtuous than their adversary is (or at least it does not seem that virtue is required for them to gain support from others).

The defining feature of underdogs likewise, as we noted earlier, is the opposition they are facing. They struggle against entities, which are fundamentally akin to themselves but significantly mightier and thus a numerical quantification of the disadvantage can be roughly generated if not outright computed. While heroes may face their kind under daunting circumstances, they also often struggle with powerful systems attempting to suppress them (e.g., authoritarian rule, racial bias, gender perceptions) or forces of nature (e.g., terminal disease, major disability, a perfect storm).

This emphasis on great disadvantage is in full manifestation when partisans attempt to marshal world public opinion in support of their cause. For example, Dr. Alan Dershowitz, the noted legal scholar and a great supporter of the state of Israel fights hard against the notion that the Palestinians are the underdog in this protracted and bloody conflict. In his book “The case for Israel” (Dershowitz, 2003), he writes, “Viewed from a global perspective, Israel is clearly the underdog. The Palestinians have the widespread support of a billion Muslims. Add to that the United Nations, the European community, the third world, the Vatican, many influential academics, the international left, the far right and many Protestant churches. The Palestinians have far more support than the Tibetans, the Kurds, the Armenians, the Chechens and many real underdogs. Moreover the nations that are oppressing these other underdog groups-China, Turkey and Russia, are far more powerful than tiny Israel, with the population of approximately 5.37 million Jews and 1.26 million Arabs. Yet these other “underdogs” receive little support from those who champion the Palestinians” (pp. 213–4). Naturally, Dr. Dershowitz picks and chooses selectively his domains of comparisons in order to garner support for the Jewish state as one can easily point to its vast superiority in terms of military might, financial resources and its geographical size (Vandello et al., 2007) and population.

In sum, we find that in a direct, two-sided, zero-sum competition when the disparity between the contestants is noticeably large, participants gravitate cross-culturally toward the lesser entity, hoping for it to prevail. At the root of this tendency, as Jesus identified early on, is the big gap in resource allocation, which is not intuitively attributed to recklessness by the underdog, but possibly to a fundamentally unjust world and thus the rejection of the privileged. In-line, fairness and competence are seen as important inferences propelling support for those who take on the mighty.

## Ethics Statement

This study was carried out in accordance with the recommendations of “name of guidelines, name of committee” with written informed consent from all subjects. All subjects gave written informed consent in accordance with the Declaration of Helsinki. The protocol was approved by the “name of committee.”

## Author Contributions

NG and YG conceived the study and drafted the manuscript. NG designed the study, supervised the data collection, and carried out the statistical analyses. KK edited the manuscript.

## Conflict of Interest Statement

The authors declare that the research was conducted in the absence of any commercial or financial relationships that could be construed as a potential conflict of interest. The reviewer AG and handling Editor declared their shared affiliation.
